# L-2hydroxyglutaric acid rewires amino acid metabolism in colorectal cancer via the mTOR-ATF4 axis

**DOI:** 10.1038/s41388-023-02632-7

**Published:** 2023-03-06

**Authors:** Sho Tabata, Yasushi Kojima, Takeharu Sakamoto, Kaori Igarashi, Ko Umetsu, Takamasa Ishikawa, Akiyoshi Hirayama, Rie Kajino-Sakamoto, Naoya Sakamoto, Ken-ichi Yasumoto, Keiichi Okano, Yasuyuki Suzuki, Shinichi Yachida, Masahiro Aoki, Tomoyoshi Soga

**Affiliations:** 1 Institute for Advanced Biosciences, Keio University, Tsuruoka, Yamagata, 997-0052 Japan; 2 Institute for Protein Research, Osaka University, Suita, Osaka, 565-0871 Japan; 3 Division of Pathophysiology, Aichi Cancer Center Research Institute, Nagoya, Aichi 464-8681 Japan; 4 Department of Cancer Biology, Institute of Biomedical Science, Kansai Medical University, Hirakata, Osaka 573-1010 Japan; 5 Department of Molecular Pathology, Graduate School of Biomedical and Health Sciences, Hiroshima University, Hiroshima, 734-8551 Japan; 6 Department of Molecular and Chemical Life Sciences, Graduate School of Life Sciences, Tohoku University, Sendai, Miyagi 980-8578 Japan; 7 Gastroenterological Surgery, Faculty of Medicine, Kagawa University, Miki-cho, Kagawa 761-0793 Japan; 8 Hyogo Prefectural Awaji Medical Center, Sumoto, Hyogo 656-0021 Japan; 9 Department of Genomic Medicine, National Cancer Center Research Institute, Chuo-ku, Tokyo, 104-0045 Japan; 10 Department of Cancer Genome Informatics, Graduate School of Medicine, Osaka University, Suita, Osaka, 565-0871 Japan; 11 Department of Cancer Physiology, Nagoya University Graduate School of Medicine, Nagoya, Aichi 466-8550 Japan

**Keywords:** Cancer metabolism, Nutrient signalling

## Abstract

Oncometabolites, such as D/L-2-hydroxyglutarate (2HG), have directly been implicated in carcinogenesis; however, the underlying molecular mechanisms remain poorly understood. Here, we showed that the levels of the L-enantiomer of 2HG (L2HG) were specifically increased in colorectal cancer (CRC) tissues and cell lines compared with the D-enantiomer of 2HG (D2HG). In addition, L2HG increased the expression of ATF4 and its target genes by activating the mTOR pathway, which subsequently provided amino acids and improved the survival of CRC cells under serum deprivation. Downregulating the expression of L-2-hydroxyglutarate dehydrogenase (L2HGDH) and oxoglutarate dehydrogenase (OGDH) increased L2HG levels in CRC, thereby activating mTOR-ATF4 signaling. Furthermore, L2HGDH overexpression reduced L2HG-mediated mTOR-ATF4 signaling under hypoxia, whereas L2HGDH knockdown promoted tumor growth and amino acid metabolism in vivo. Together, these results indicate that L2HG ameliorates nutritional stress by activating the mTOR-ATF4 axis and thus could be a potential therapeutic target for CRC.

## Introduction

Cancer cells exhibit distinct metabolic rewiring compared to normal cells as they have adapted to a harsh microenvironment characterized by hypoxia and a minimal nutrient supply. D/L-2-hydroxyglutaric acid (2HG) is a characteristic metabolite of cancer cells that has been shown to directly contribute toward the malignant progression of cancer, and has thus attracted considerable attention as an oncometabolite.

Since 2008, several specific genetic mutations have been discovered in isocitrate dehydrogenase (IDH), an enzyme in the citric acid cycle, in brain tumors and leukemias [1, 2]. It was subsequently found that mutant IDH induces carcinogenesis by allowing cells to produce the D-enantiomer of 2HG (D2HG) [3, 4], which promotes cancer progression by inhibiting the function of α-ketoglutarate (αKG)-dependent enzymes, such as proline hydroxylases, histone demethylases (KDM), and DNA hydroxymethylases (TET family) [5, 6]. Furthermore, recent reports have indicated that D2HG is involved in metabolic responses, signal transduction, and genomic integrity [7–12].

In contrast, it has been reported that the levels of the L-enantiomer of 2HG (L2HG) are increased by changes in the tumor microenvironment, including hypoxia and a low pH [13–16]. The mechanisms underlying this increase in L2HG levels include an increase in the expression or activity of lactate dehydrogenase A (LDHA) and malate dehydrogenase (MDH), and a decrease in the expression or activity of L2HG dehydrogenase (L2HGDH) [13–16]. Notably, L2HG is important for adaptation to hypoxic stress and may be involved in redox homeostasis and the activity of the hypoxia-inducible factor-1 pathway [17]. Although L2HG and D2HG have been predicted to have many similar functions, L2HG has higher lysine demethylase (KDM) and ten-eleven translocation (TET) inhibitory activities than D2HG [5] and the underlying molecular mechanisms are not yet fully understood.

Previously, we performed a comprehensive metabolic analysis and identified specific metabolic changes in human colorectal cancer (CRC), including increased levels of S-adenosylmethionine, a donor molecule for DNA and histone methylation, decreased glucose levels [18], and significantly increased 2HG levels in CRC tissues without *IDH* mutation. However, the roles of 2HG and the mechanisms underlying its accumulation in CRC remain unclear. In this study, we identified a specific increase in L2HG levels in CRC and investigated its biological significance and the underlying molecular mechanisms.

## Results

### L2HG levels are increased in colorectal tumors

**Fig. 1:**
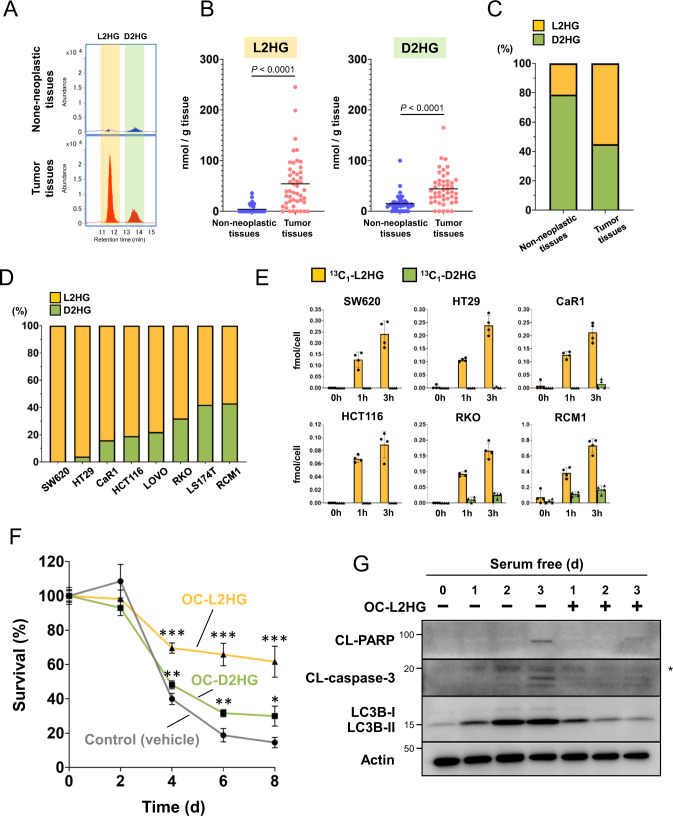
L2HG levels are increased in colorectal tumors. **A**, **B** Representative chromatograms (**A**) and levels (**B**) of L2HG and D2HG in paired non-neoplastic and CRC tissues obtained from 47 patients with CRC. **C** Ratio of L2HG and D2HG in non-neoplastic and CRC tissues. **D** Ratio of L2HG and D2HG in CRC cell lines. **E** ^13^C_1_-labeled L2HG and D2HG in CRC cell lines. Data represent the mean ± SD of four independent experiments. **F** Viability of CaR1 cells treated with OC-L2HG (250 μM) or OC-D2HG (250 μM) under serum-free conditions. Viability was assessed using MTT assays. Data represent the mean ± SD of three independent experiments. *P < 0.05, **P < 0.01, and ***P < 0.001. **G** Expression of CL-PARP, CL-caspase 3, and LC3B in CaR1 cells treated with OC-L2HG under serum-free conditions. *non-specific band.

To determine the relative contribution of the two 2HG enantiomers in CRC, we quantified their expression using liquid chromatography-time of flight mass spectrometry (LC-TOFMS). We found that the levels of both 2HG isomers were upregulated in CRC tumor tissues compared to non-neoplastic tissues (Fig. 1A, B). In particular, L2HG levels were very low in non-neoplastic tissues but were markedly increased in CRC tumors (Fig. 1C). L2HG levels were consistently higher than those of D2HG in the eight CRC cell lines tested (Fig. 1D). Furthermore, ^13^C-flux analysis using ^13^C_1_-glutamine (^13^C_1_-Gln) revealed that ^13^C-labeled L2HG was produced at a much higher rate than ^13^C-labeled D2HG in most CRC cell lines (Fig. 1E).

Previous studies have demonstrated that 2HG contributes toward cell survival under serum-free stress conditions [19, 20]. Therefore, we examined the effects of the two 2HG enantiomers on survival of CaR1 and HT29 cells. When cells were treated with membrane-permeable octyl esters of L2HG (OC-L2HG) under serum-free stress, cell survival was significantly higher than when treated with membrane-permeable octyl esters of D2HG (OC-D2HG; Fig. 1F; Supplementary Fig. S1A, B). In addition, L2HG suppressed the expression of markers of apoptosis (CL-PARP and CL-caspase 3) and autophagy (LC3B-II; Fig. 1G; Supplementary Fig. S1C). Together, these findings suggest that L2HG production is upregulated in CRC tumors and contributes toward cell survival.

### L2HG induces the expression of ATF4 and its target genes in CRC cells

**Fig. 2:**
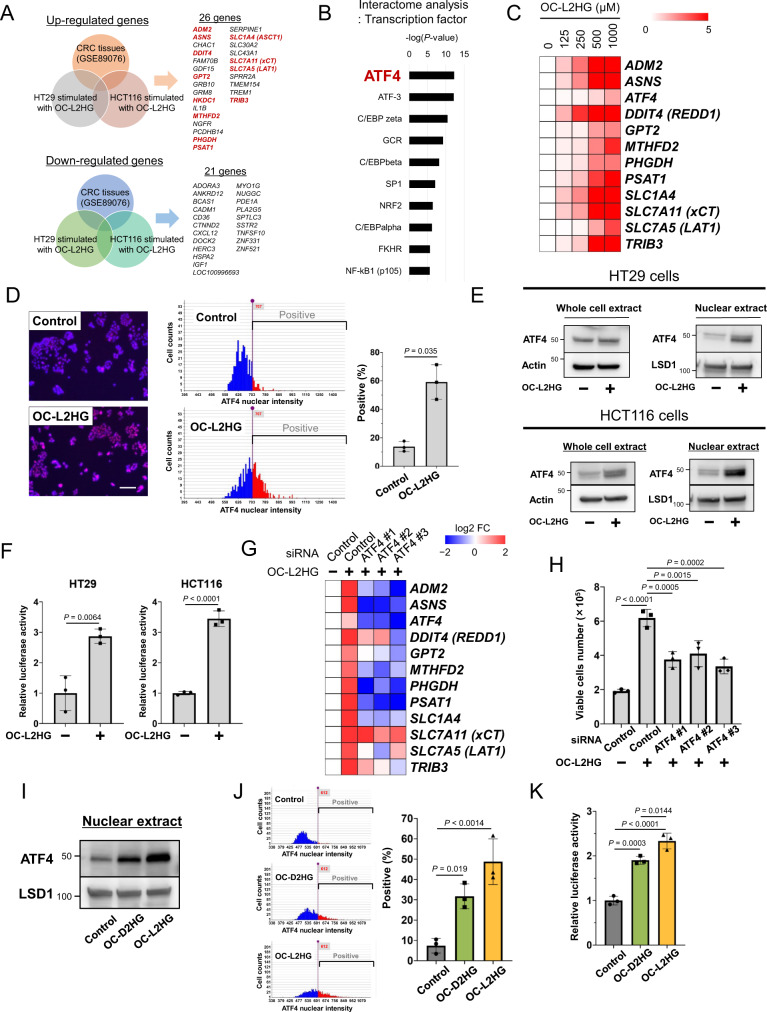
L2HG induces ATF4 activation in CRC cells. **A** Common up- or down-regulated genes in CRC tissues and OC-L2HG-stimulated HT29 and HCT116 cells. HT29 and HCT116 cells were treated with 500 μM OC-L2HG for 24 h. **B** Interactome analysis of transcription factors among the 26 genes upregulated by OC-L2HG. **C** Analysis of the mRNA expression of *ATF4* and ATF4 target genes in HT29 cells treated with OC-L2HG for 24 h, using real-time PCR. Heat map showing the gene expression levels of cells stimulated with the indicated concentrations of OC-L2HG as a scaling ratio relative to unstimulated cells. **D** Immunofluorescence staining and quantification of ATF4 in HT29 cells treated with 500 μM OC-L2HG. Data represent the mean ± SD of three independent experiments. Scale bar, 200 μm. **E** ATF4 protein expression in the whole and nuclear fractions of HT29 and HCT116 cells treated with 500 μM OC-L2HG. **F** Transcriptional activity of ATF4 in HT29 and HCT116 cells treated with 500 μM OC-L2HG (see Methods). Data represent the mean ± SD of three independent experiments. **G** Effect of *ATF4* knockdown on the mRNA expression of ATF4 target genes in HT29 cells treated with 500 μM OC-L2HG. mRNA levels in each sample were converted to a log2 fold-change relative to the control. Red and blue indicate higher and lower levels, respectively, compared to the control (white). **H** Effect of *ATF4* knockdown on the viability of CaR1 cells treated with L2HG (250 μM) under serum-free conditions for 6 d. Viable cells were counted using a trypan blue exclusion assay. Data represent the mean ± SD of three independent experiments. **I**, **J** Expression levels of nuclear ATF4 in HT29 cells treated with 500 μM OC-L2HG or 500 μM OC-D2HG for 24 h. Nuclear ATF4 expression was measured using western blotting (**I**) and immunofluorescence staining (**J**). Data represent the mean ± SD of three independent experiments. **K** Transcriptional activity of ATF4 in HT29 and HCT116 cells treated with OC-L2HG or OC-D2HG. Data represent the mean ± SD of three independent experiments.

To elucidate the molecular mechanisms through which L2HG contributes toward cell survival, we performed transcriptome analysis in L2HG-treated CRC cells and using data previously obtained from CRC tissues [18]. A total of 26 and 21 genes were up- and downregulated, respectively (Fig. 2A). Interaction analysis for transcription factors revealed that the upregulated genes were enriched for activating transcription factor 4 (ATF4) target genes (e.g., ADM2, DDIT4, ASNS, and SLC7A5; Fig. 2B). Furthermore, L2HG increased the expression of ATF4 target genes in HT29 and HCT116 cells in a dose- and time-dependent manner (Fig. 2C; Supplementary Fig. S2A–C), as well as ATF4 mRNA expression. The levels of ATF4 target genes were also upregulated by L2HG in CaR1 cells (Supplementary Fig. S2D).

Next, we focused on the mechanism of ATF4 activation by L2HG in CRC cells (Fig. 2D–H). L2HG increased ATF4 expression in both the whole and nuclear fractions of CRC cells (Fig. 2D, E) and enhanced the activity of the ATF4-responsive reporter (Fig. 2F). Conversely, siRNA-mediated *ATF4* knockdown suppressed the induction of ATF4 target genes by L2HG in CRC cells (Fig. 2G; Supplementary Fig. S2E, F) and abrogated the effect of L2HG on cell survival under serum-free stress (Fig. 2H). Thus, L2HG appears to contribute toward the survival of CRC cells by promoting the expression of ATF4 and its target genes.

When we further examined the ability of 2HG enantiomers to induce ATF4 expression, we found that L2HG induced the expression of ATF4 and its target genes more strongly than D2HG after ATF4 nuclear translocation and ATF4-dependent transcription (Fig. 2I–K; Supplementary Fig. S2G).

### L2HG increases ATF4 expression via mTOR signaling in CRC cells

**Fig. 3:**
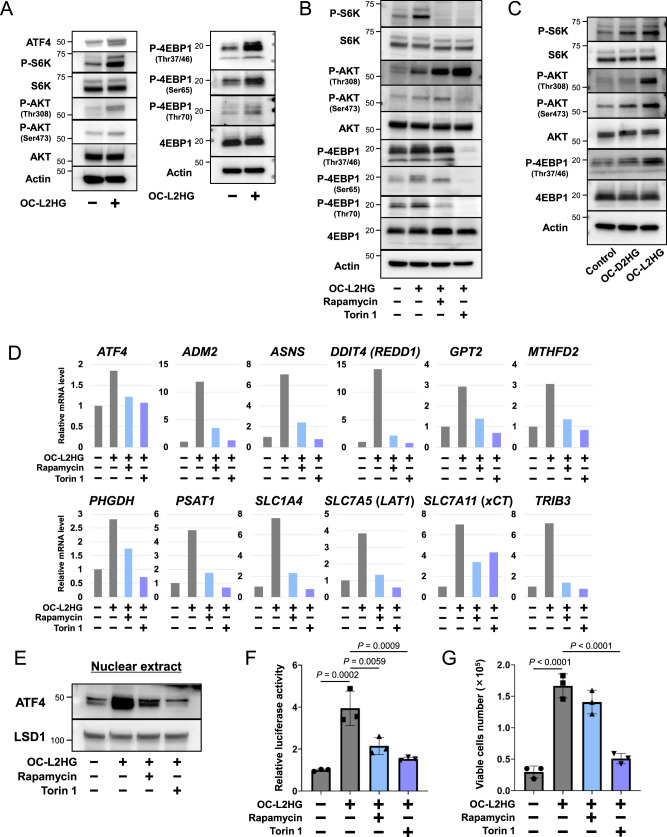
L2HG increases ATF4 expression via mTOR signaling in CRC cells. **A** Representative western blot analyses of signaling components in the mTOR pathway. HT29 cells were treated with 500 μM OC-L2HG for 24 h under serum-free conditions (see Methods). **B** Effect of rapamycin (mTORC1 inhibitor) and Torin1 (mTORC1 and mTORC2 inhibitor) on the phosphorylation of S6K, AKT, and 4EBP1 in HT29 cells. Cells were treated with 500 μM OC-L2HG, 100 nM rapamycin, and/or 250 nM Torin1 for 24 h under serum-free conditions. **C** Differences between 2HG enantiomers in the phosphorylation of S6K, AKT, and 4EBP1 in HT29 cells. **D**–**F** Effect of rapamycin and Torin1 on the mRNA expression of ATF4 target genes (**D**), the expression of nuclear ATF4 (**E**), and the transcriptional activity of ATF4 (**F**). qPCR data are the average of three technical replicates and are representative of at least two independent experiments with similar results. **G** Effect of rapamycin and Torin1 on the viability of CaR1 cells treated with L2HG (250 μM) under serum-free conditions for 6 d. Viable cells were counted using a trypan blue exclusion assay. Data represent the mean ± SD of three independent experiments.

Mammalian target of rapamycin complex 1 (mTORC1) has been shown to increase ATF4 expression by enhancing the stability and translation of its mRNA [21–23]. Therefore, we investigated whether mTOR signaling is involved in L2HG-mediated ATF4 induction in CRC cells. L2HG enhanced the phosphorylation of mTORC1 downstream effectors, p70 ribosomal S6 kinase (S6K), and 4E-binding protein (4EBP1; Fig. 3A), as well as the phosphorylation of AKT Ser473 (downstream of mTORC2) and AKT Thr308 (upstream of mTORC2; Fig. 3A). However, L2HG did not alter the expression of DEP domain-containing mTOR-interacting protein (DEPTOR), a negative regulator of mTORC1/2 (Supplementary Fig. S3A). Rapamycin (an mTORC1 inhibitor) and Torin1 (an mTORC1 and mTORC2 inhibitor) suppressed the L2HG-mediated phosphorylation of S6K and 4EBP1 (Fig. 3B). Furthermore, Torin1 decreased the L2HG-mediated phosphorylation of AKT Ser473, whereas rapamycin and Torin1 increased the phosphorylation of AKT Thr308, likely due to the feedback activation of upstream signaling (Fig. 3B). Notably, L2HG induced the phosphorylation of S6K, 4EBP1, and AKT more potently than D2HG (Fig. 3C).

Next, we examined the effect of rapamycin and Torin1 on ATF4 induction in CRC cells (Fig. 3D–F), finding that both inhibitors suppressed the L2HG-mediated induction of ATF4 target genes (Fig. 3D), ATF4 nuclear localization (Fig. 3E), and ATF4-dependent transcription (Fig. 3F). Remarkably, both inhibitors downregulated ATF4 expression (Fig. 3D) but only Torin1 significantly inhibited L2HG-mediated cell survival advantages under serum starvation stress (Fig. 3G).

2HG is known to be a competitive inhibitor of αKG-dependent dioxygenases (αKGDs) [17]. We examined whether exogenous αKG could override the effect of L2HG and prevent activation of mTOR/ATF4. When cells were treated with membrane-permeable octyl esters of αKG (OC-αKG), the inductions of S6K phosphorylation and ATF4 expression by L2HG were downregulated (Supplementary Fig. S3B). L2HG-induced ATF4 target genes expression decreased as well (Supplementary Fig. 3C). Together, these results suggest that L2HG increases the expression and transcriptional activity of ATF4 via mTOR signaling in dependence of the inhibitory activity of αKGDs.

### L2HG mediates ATF4 induction to regulate amino acid metabolism in CRC cells

**Fig. 4:**
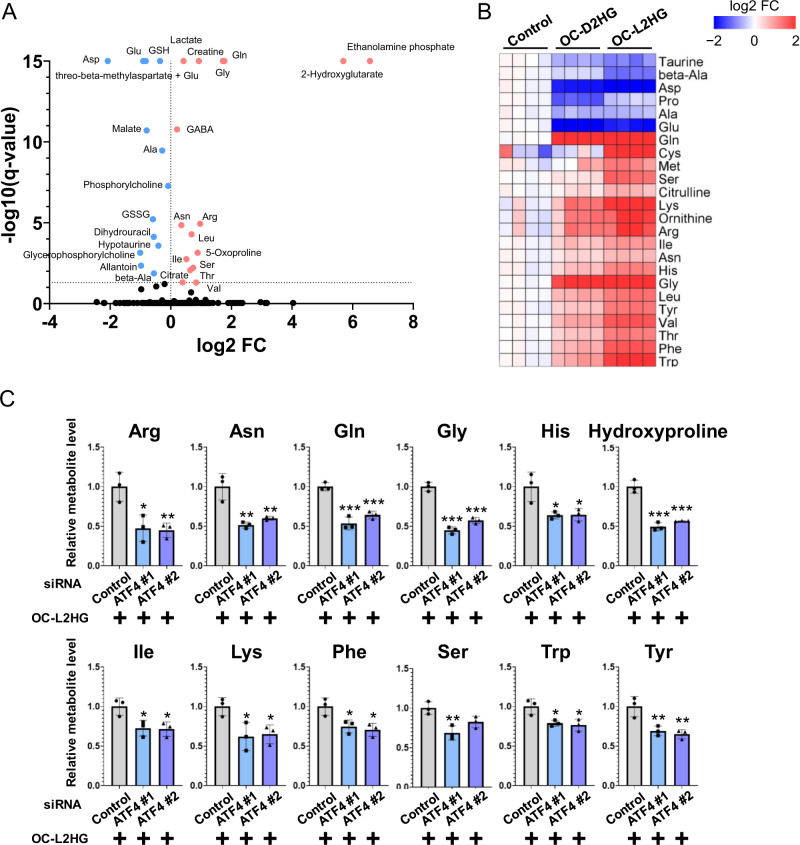
L2HG mediates ATF4 induction to regulate amino acid metabolism in CRC cells. **A** Volcano plot showing the levels of metabolites that were significantly altered in HT29 cells treated with 500 μM OC-L2HG for 24 h. Red and blue dots indicate significantly up- and down-regulated metabolites, respectively. Metabolites levels were detected by capillary electrophoresis time-of-flight mass spectrometry. **B** Effect of OC-L2HG and OC-D2HG on amino acid levels in HT29 cells. Amino acid levels in each sample were converted to a log2 fold-change relative to the control. Red and blue indicate higher and lower levels, respectively, as compared to the control (white). **C** Effect of *ATF4* knockdown on amino acid levels in HT29 cells stimulated with OC-L2HG. Data represent the mean ± SD of three independent experiments.

ATF4 is a critical transcription factor for amino acid metabolism that induces the expression of various amino acid transporters and enzymes (e.g., SLC7A5, SLC7A11, ANSN, and PHGDH) and contributes toward metabolic properties, including redox balance, autophagy, energy production, and nucleotide synthesis. To determine whether L2HG affects amino acid metabolism via ATF4 activation, we performed metabolome analysis in CRC cells treated with OC-L2HG (Supplementary Table 1). L2HG significantly altered the levels of 23 metabolites, including many amino acids (Fig. 4A, B). As expected, L2HG increased the levels of Tyr, Leu, Ile, Val, Phe, and Cys, which are amino acids imported through SLC7A5 or SLC7A11 [24], increased the levels of Ser and Gly, which are synthesized by PHGDH and PSAT1 [25], and elevated the ratio of Asn to Asp, which is controlled by ASNS (Fig. 4B; Supplementary Fig. S4A, B). We also examined the effect of the two 2HG enantiomers on amino acid metabolism and found that L2HG had a larger impact than D2HG, consistent with the observed ATF4 induction (Fig. 4B). In contrast, *ATF4* knockdown decreased the levels of amino acids upregulated by L2HG (Fig. 4C). These results suggest that L2HG rewires amino acid metabolism by inducing ATF4.

### L2HGDH and OGDH downregulation increase L2HG levels and induce ATF4 in CRC

**Fig. 5:**
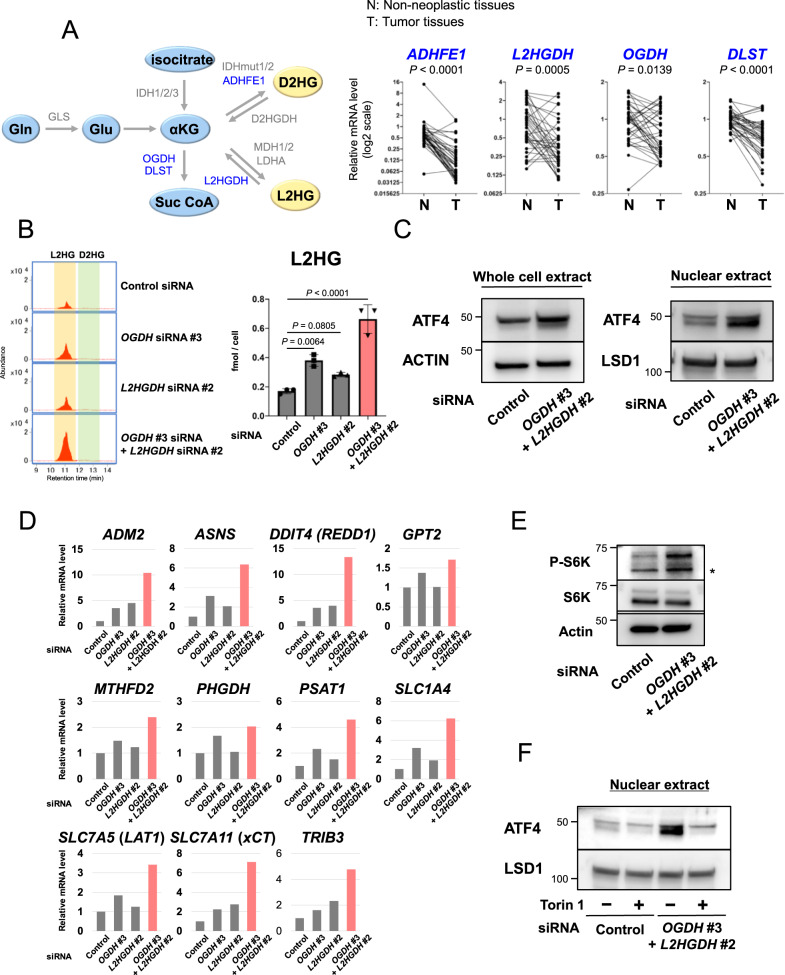
L2HGDH and OGDH downregulation increase L2HG levels and induce ATF4 in CRC. **A** Schematic pathway of L2HG and D2HG production (left) and the mRNA expression of *ADHFE1*, *L2HGDH*, *OGDH*, and *DLST* in paired non-neoplastic and CRC tissues (right). mRNA levels were measured using microarray analysis. **B** Representative chromatograms (left) and levels (right) of L2HG in HT29 cells transfected with *OGDH* and/or *L2HGDH* siRNA. Data represent the mean ± SD of three independent experiments. **C** ATF4 protein expression in the whole and nuclear fractions of *OGDH* and *L2HGDH* knockdown HT29 cells. **D** Effect of *OGDH* and *L2HGDH* knockdown on the mRNA expression of ATF4 target genes in HT29 cells. mRNA levels were measured using real-time PCR. Data are the average of three technical replicates and are representative of at least two independent experiments with similar results. **E** S6K phosphorylation in *OGDH* and *L2HGDH* knockdown HT29 cells. **F** Effect of Torin1 (250 nM) on the expression of nuclear ATF4 in *OGDH* and *L2HGDH* knockdown HT29 cells.

Next, we investigated the mechanism underlying increased L2HG levels in CRC cells. Among the metabolic enzymes involved in D2HG and L2HG production (Fig. 5A) [19, 26–29], *L2HGDH*, *ADHFE1*, *OGDH*, and *DLST* were downregulated in CRC tissues from our transcriptome data (Fig. 5A, right) [18]. Notably, the siRNA-mediated knockdown of *L2HGDH* or *OGDH*, but not D2HG, upregulated L2HG in CRC cells (Fig. 5B; Supplementary Fig. S5A–D), with their combined knockdown further enhancing L2HG induction (Fig. 5B). We also observed that the knockdown of *L2HGDH* and *OGDH* elevated the expression of ATF4 target genes, the levels of ATF4 in whole-cell and nuclear extracts, and the phosphorylation of S6K (Fig. 5C–E; Supplementary Fig. S5C, D); however, Torin1 abrogated the induction of ATF4 (Fig. 5F). These results indicate that downregulating L2HGDH and OGDH in CRC cells is sufficient to induce L2HG and thus ATF4 by activating mTOR signaling.

**Fig. 6:**
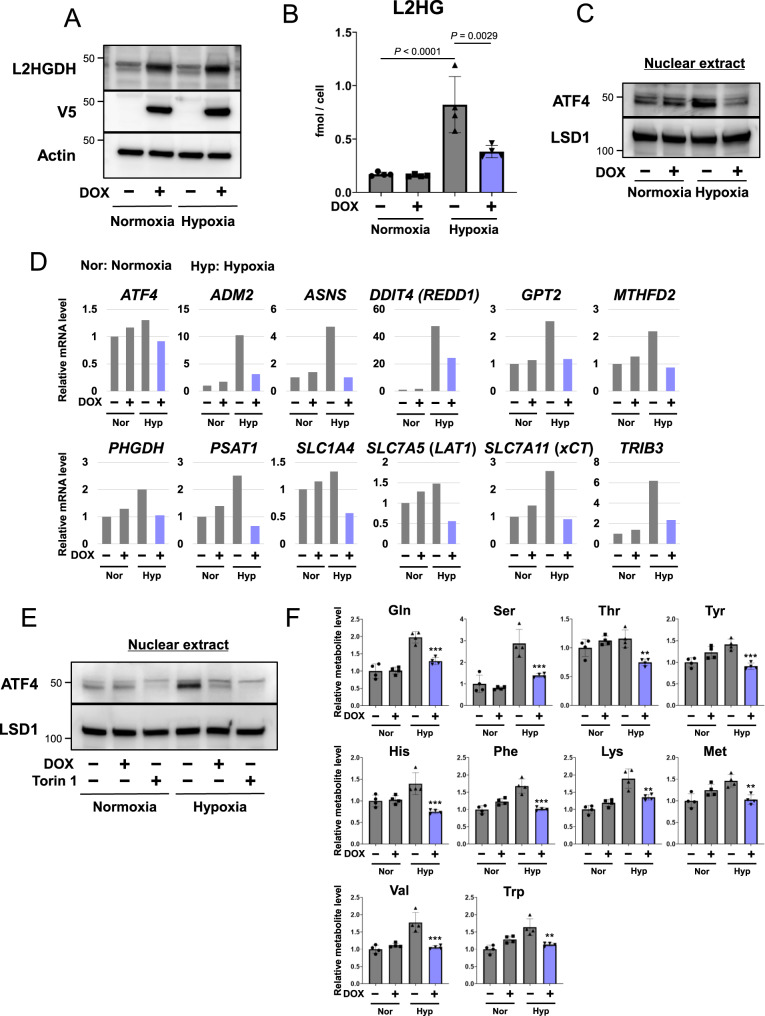
Ectopic L2HGDH expression suppresses L2HG levels and induces ATF4 under hypoxic conditions. **A** Expression of doxycycline (DOX)-inducible V5-tagged L2HGDH in HT29 cells. Cells were treated with 1 μg/mL DOX and hypoxia (1% O_2_) for 24 h. **B**, **C** Effect of L2HGDH overexpression on the levels of L2HG (**B**) and nuclear ATF4 (**C**) in HT29 cells under hypoxic conditions. Data represent the mean ± SD of three independent experiments. **D** Effect of L2HGDH overexpression on the mRNA expression of *ATF4* and ATF4 target genes in HT29 cells under hypoxic conditions. Data are the average of three technical replicates and are representative of at least two independent experiments with similar results. **E** Effect of Torin1 (250 nM) on the hypoxic stress-induced expression of nuclear ATF4 in HT29 cells. **F** Effect of L2HGDH overexpression on amino acid levels in HT29 cells under hypoxic conditions. Data represent the mean ± SD of four independent experiments. *P < 0.05, **P < 0.01, and ***P < 0.001.

Previous studies have demonstrated that hypoxic conditions, such as those found in the tumor microenvironment, elevate L2HG levels by upregulating LDH/MDH or downregulating L2HGDH [13–16]. Therefore, we examined whether hypoxia (1% O_2_)-mediated L2HG upregulation activates ATF4 in CRC cells. Culturing CRC cells under hypoxic stress for 24 h upregulated the levels of L2HG, nuclear ATF4, and ATF4 target genes (Fig. 6A–E). Conversely, doxycycline (DOX)-inducible L2HGDH expression suppressed these hypoxia-induced effects (Fig. 6A–E) and Torin1 decreased hypoxia-induced ATF4 accumulation in the nuclear fraction (Fig. 6E). In addition, L2HGDH overexpression decreased the levels of several amino acids under hypoxic conditions (Fig. 6F). Taken together, these results indicate that hypoxia-mediated L2HG induction activates mTOR-ATF4 signaling in CRC cells.

### *L2HGDH* knockdown increases tumor growth and alters amino acid metabolism in vivo

**Fig. 7:**
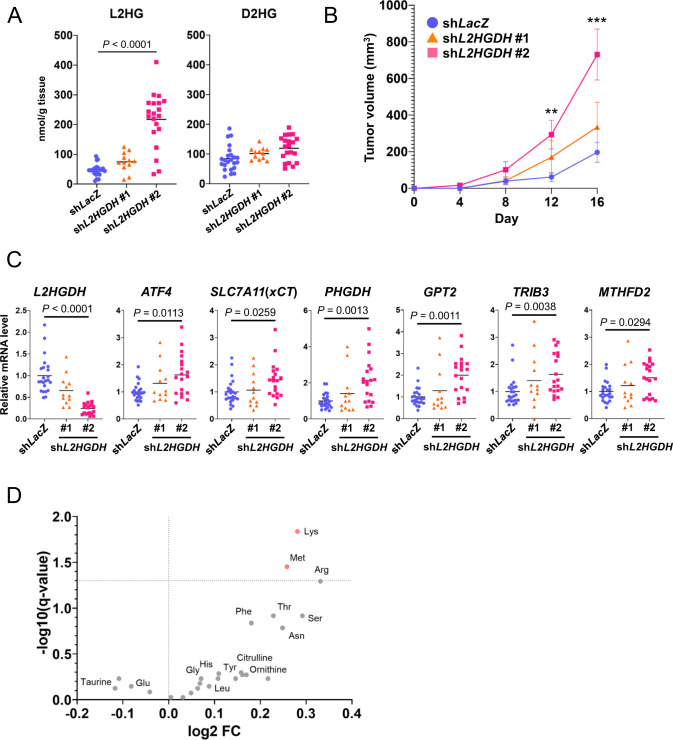
L2HGDH knockdown increases tumor growth and alters amino acid metabolism in vivo. **A** L2HG and D2HG levels in tumors derived from control (sh*LacZ*, *n* = 23), *L2HGDH-*knockdown (sh*L2HGDH* #1, *n* = 12; sh*L2HGDH* #2, *n* = 20) HT29 cells. **B** Rate of growth following subcutaneous implantation of sh*LacZ* (*n* = 23), sh*L2HGDH* #1 (*n* = 12), and sh*L2HGDH* #2 (*n* = 21) HT29 cells. Data represent the mean ± SEM. **P < 0.01 and ***P < 0.001. **C** mRNA expression of *L2HGDH*, *ATF4*, and ATF4 target genes in tumors derived from sh*LacZ* (*n* = 23), sh*L2HGDH* #1 (*n* = 12), and sh*L2HGDH* #2 (*n* = 19) HT29 cells. **D** Volcano plot showing the levels of amino acids that were significantly altered in in tumors derived from sh*LacZ* (*n* = 16) and sh*L2HGDH* #2 (*n* = 14) HT29 cells. Red dots indicate significantly upregulated amino acids. Levels of amino acids were detected using capillary electrophoresis time-of-flight mass spectrometry.

**Fig. 8:**
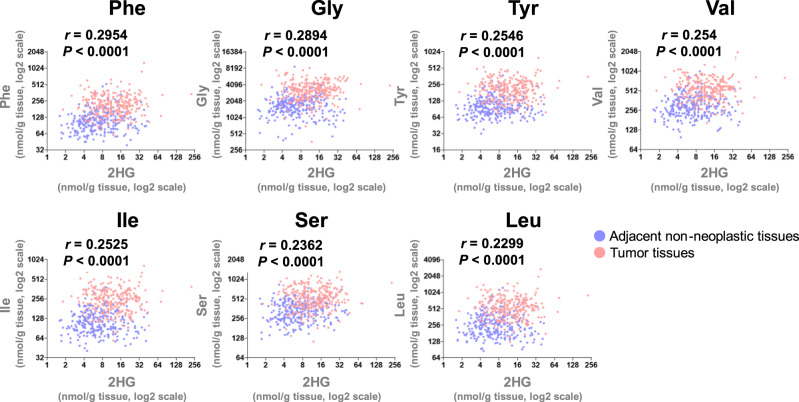
Amino acids and 2HG levels in human CRC tumors. Correlation between amino acids (Phe, Gly, Tyr, Val, Ile, Ser, and Leu) and 2HG levels in paired tumors and adjacent non-neoplastic tissues from 275 CRC patients. The metabolites levels were measured using capillary electrophoresis time-of-flight mass spectrometry.

To investigate the function of L2HG in vivo, we evaluated the effect of *L2HGDH* knockdown on tumor growth using a mouse xenograft model. Briefly, we subcutaneously injected *L2HGDH*-knockdown CRC cell lines transfected with *L2HGDH* shRNA#1 (sh*L2HGDH*#1) or #2 (sh*L2HGDH*#2; Supplementary Fig. S6) into nude mice. Sixteen days after injection, L2HG levels were significantly higher in sh*L2HGDH*#2 tumors than in control tumors (sh*LacZ*; Fig. 7A). Notably, the knockdown efficiency of sh*L2HGDH*#2 was higher than that of sh*L2HGDH*#1 in tumors (Fig. 7C), consistent with the increased levels of L2HG (Fig. 7A). sh*L2HGDH*#2 cells significantly increased tumor growth (Fig. 7B) as well as the expression of ATF4 target genes, including amino acid metabolism genes (Fig. 7C), and altered amino acid metabolism compared to the control (sh*LacZ*; Fig. 7D). The levels of Lys and Met were significantly increased in the L2HGDH knockdown (sh*L2HGDH*#2) tumors. Furthermore, we investigated the relationship between 2HG (sum of D2HG and L2HG) and amino acid metabolism in human CRC tumors and adjacent non-neoplastic tissues using metabolomic data presented in one of our previous studies [18] and found that levels of Phe, Gly, Tyr, Val, Ile, Ser, and Leu, regulated by ATF4 target genes such as PHGDH, SLC7A5, and SLC7A11, were significantly correlated with 2HG levels (Fig. 8). In summary, these results suggest that L2HG regulates amino acid metabolism by activating ATF4 to promote tumor growth in vivo.

## Discussion

The oncometabolites D2HG and L2HG have directly been implicated in carcinogenesis; however, the underlying molecular mechanisms remain poorly understood. Here, we demonstrated that L2HG levels were significantly increased in human CRC tissues and subsequently upregulated amino acid levels by activating the mTOR-ATF4 pathway, thereby improving cell survival under serum deprivation. In addition, we found that both L2HGDH and OGDH were downregulated in human CRC tissues, leading to increased L2HG levels and mTOR-ATF4 signaling activation. Hypoxia-induced L2HG production also contributed toward amino acid metabolism via the mTOR-ATF4 axis and *L2HGDH* knockdown in a mouse xenograft model increased L2HG levels and promoted CRC tumor growth.

Regarding L2HG concentrations in vitro and in vivo, L2HG concentrations in tissues and cultured cells cannot be simply compared because tissues include not only cells but also extracellular matrix, and normalization methods differ. L2HG concentrations averaged 54 nmol/g tissue in human colon cancer tissue (Fig. 1A) and 217 nmol/g tissue in L2HGDH knockdown tumors (Fig. 7A). Assuming a specific gravity of 1 for the tumors, these values were 60 μM and 200 μM, respectively, suggesting that the concentrations of L2HG in vitro (250 or 500 μM) were 1.2 to 8.3 times higher than those in vivo. On the other hand, the intracellular D/L-2HG concentration in HT29 cells treated with 500 μM L2HG for 24 h was approximately 6 fmol/cell (Supplementary Table 1), which was higher than the concentration induced by hypoxia (approximately 1 fmol/cell). The treatment concentration of L2HG may not reflect the biological L2HG production level and further investigation is needed. However, when endogenous L2HG production was increased by L2HGDH knockdown or hypoxia (Figs. 5 and 6), ATF4 target gene and amino acids levels increased, suggesting that the increased L2HG levels that can occur in living cells have the same effect of activating ATF4 as L2HG treatment.

We found that downregulation of L2HGDH and OGDH contributes to L2HG production and activates mTOR/ATF4 signaling in CRC cells. However, since OGDH knockdown inhibits the metabolic flux of the tricarboxylic acid cycle, the observed effect should be interpreted with caution. We thus used L2HGDH single knockdown cells for the in-vivo experiments (Fig. 7). Whether the activation of mTOR/ATF4 by OGDH knockdown fully depends on L2HG production requires further investigation.

It has previously been reported that D2HG activates the mTOR signaling pathway [30], consistent with our results. However, we found that L2HG increased the levels of P-S6K (mTORC1), P-4EBP (mTORC1), and P-AKT S473 (mTORC2) much more potently than D2HG in CRC cells under serum deprivation. D/L-2HG has also been shown to inhibit ATP synthase and mTOR signaling, and extend the life span of *C*. *elegans* [10]. Under culture conditions with abundant serum, the basal activity of mTOR was high, and further L2HG-induced activation of the mTOR signaling was not clearly observed in CRC cells. The effect of L2HG on mTOR signaling might therefore depend on nutrient conditions. In addition, the effect of L2HG might vary among different cell types, species, or genetic backgrounds.

D2HG has been reported to activate mTOR signaling by inhibiting KDM4, an αKG-dependent enzyme of the Jumonji family of lysine demethylases, which is involved in the protein stability of DEPTOR, a negative regulator of mTORC1/2 [30]. However, we found that DEPTOR expression was not altered by L2HG in CRC cells (Supplementary Fig. S3A), suggesting that other pathways might be involved. Notably, L2HG increased the amino acid pool (Supplementary Fig. S4), including several amino acids (Gln, Val, Leu, Met, and Arg) that are known to activate mTOR signaling [31, 32], suggesting the existence of a positive feedback mechanism. Further studies are needed to fully determine the relationship between mTOR signaling and L2HG in CRC cells.

ATF4, a member of the CREB/ATF family of bZIP transcription factors, can regulate both pro-survival and pro-apoptotic signaling pathways [33, 34] and plays a critical role in various biological processes, including the redox response, amino acid metabolism, autophagy, senescence, and apoptosis. The functions of ATF4 depend on the cell type, nutrient conditions, stressor properties, and the duration of stress. Although previous studies have shown that ATF4 is a crucial mediator of the integrative stress response (ISR) that is controlled by the phosphorylation of the translation factor eIF2α [33, 34], emerging evidence has suggested that mTORC1 signaling also activates ATF4 by regulating its mRNA translation and stability [21–23]. Importantly, the mTORC1-ATF4 pathway also increases the expression of specific ATF4 target genes, including those involved in amino acid uptake, synthesis, and tRNA charging, compared to the wide range of genes expressed during ISR-induced ATF4 activation [35]. In this study, we found that the L2HG-induced activation of mTOR-ATF4 signaling contributed toward altered amino acid metabolism and survival under serum deprivation in CRC. Moreover, L2HG suppressed autophagy in CRC cells cultured under serum-free conditions for three days (Fig. 1G; Supplementary Fig. S1C). Since mTORC1 activation and amino acid supply abrogate autophagy [36, 37], L2HG could be an essential regulator of autophagy. In addition, a recent study showed that mitochondrial stress can cause neuronal dysfunction through ATF4-dependent increases in L2HG levels in *Drosophila* brains [38]. Together with our findings, these studies suggest the existence of bidirectional feedback between L2HG and ATF4, which might have biological significance in CRC and the nervous system.

In RCC, decreased L2HGDH levels have been reported to lead to L2HG accumulation, contributing toward cancer malignancy [39–41]. In addition, the loss of heterozygosity of *L2HGDH*, located on chromosome 14q, was shown to be correlated with downregulated L2HGDH expression [39, 40]. High L2HG levels have also been observed in metastatic RCC tumors, conferring a poor prognosis. Here, we found that ectopic L2HGDH expression reduced intracellular L2HG levels and inhibited CRC tumor growth in vivo. Recently, an L2HG-sensing FRET sensor was developed to evaluate the biological functions of L2HGDH and hypoxia-induced L2HG accumulation [42]. Although the functions of L2HG have been reported in RCC, its roles in other types of cancer remain largely unknown. Our study revealed the pathological significance of L2HG in CRC and proposed that L2HG regulates amino acid metabolism through the mTOR-ATF4 axis.

Furthermore, we found that both L2HG and D2HG were upregulated in human CRC tumor tissues (Fig. 1A), consistent with previous reports that urinary D2HG levels correlate positively with the number of subsequent polyps and dysplasia severity in a mouse model of colitis-associated CRC [43] and that D2HG contributes toward epithelial-mesenchymal transition and tumor metastasis in CRC [44]. However, L2HG levels increased more in tumors than D2HG and L2HG was a more potent mediator of mTOR-ATF4 signaling activation. Thus, L2HG and D2HG may act synergistically as oncometabolites to mediate the malignant phenotype of CRC and could serve as targets for CRC therapy.

## Materials and methods

### Reagents and cell culture

OC-L2HG, OC-D2HG, and OC-αKG were purchased from Toronto Research Chemicals (Toronto, Canada). ^13^C^1^-Gln was purchased from Cambridge Isotope Laboratories, Inc. (Tewksbury, MA, USA). Rapamycin and Torin1 were purchased from LC Laboratories (Woburn, MA, USA) and Merck Millipore (Burlington, MA, USA), respectively. DMOG was purchased from Sigma-Aldrich (St Louis, MO, USA).

The CRC cell lines, HT29, HCT116, and SW620, were obtained from the American Type Culture Collection (ATCC; Manassas, VA, USA). LOVO, CaR1, and RCM1 cells were obtained from the Japanese Collection of Research Bioresources (JCRB; Osaka, Japan). The RKO cell line was a gift from Dr. M. Tsujii at Osaka University (Osaka, Japan). All cells were grown in RPMI 1640 medium (Wako Pure Chemical Industries, Ltd., Osaka, Japan) containing 10% (v/v) fetal bovine serum (FBS; Sigma-Aldrich), and antibiotics (100 U/mL penicillin, 100 mg/mL streptomycin, and 0.25 mg/mL amphotericin B; Nakarai Tesque, Kyoto, Japan) at 37 °C in a humidified atmosphere with 5% CO_2_.

### Animal care

All animal experiments were carried out according to protocols approved by the Animal Care and Use Committee of Aichi Cancer Center Research Institute (Nagoya, Japan). BALB/c nu/nu mice were purchased from CLEA Japan (Tokyo, Japan) and acclimated for at least one week before any experimental procedures. Mice were housed in a specific-pathogen-free facility at room temperature with standard day-night cycles and were provided with commercial laboratory chow (CLEA Rodent Diet CE-2; CLEA Japan, Tokyo, Japan) and autoclaved tap water ad libitum.

### Tumor growth assay

Six-week-old female BALB/c nu/nu mice were subcutaneously injected with 1 × 10^6^ HT29 cells expressing shRNA against *L2HGDH* or *LacZ* under anesthesia (medetomidine hydrochloride (0.75 mg/kg), midazolam (4 mg/kg), butorphanol tartrate (5 mg/kg). The implanted tumors were measured using a caliper on the indicated days and their volumes were calculated using the following formula: V = (L × W^2^) / 2, where V is the volume (mm^3^), L is the largest tumor diameter (mm), and W is the smallest tumor diameter (mm).

### Human colorectal tumor samples for D- and L-2HG measurements

As described previously [18], we conducted all experiments according to a study protocol approved by the Institutional Ethics Committee of Kagawa University (Heisei 24–040) after obtaining informed consent from all subjects. Tumor and surrounding non-neoplastic tissues were surgically obtained from 47 colorectal cancer patients as described previously [18] (Supplementary Table 2).

### Analysis of D- and L-2HG

LC-TOFMS was performed using an Agilent 1200 series HPLC system (Agilent Technologies, Palo Alto, CA, USA) and an Agilent 6220 TOFMS. D- and L-2HG were separated using an Astec CHIROBIOTIC R column (2.1 mm i.d. × 250 mm, 5 µm; Supelco, Bellefonte, PA, USA) that was maintained at 20 °C. Isocratic elution was performed using 75% ethanol/methanol (3:1, v/v) and 25% water containing 0.1% trimethylamine acetate (pH 4.5) as the mobile phase at a flow rate of 0.1 mL/min. The sample injection volume was 1 µL. TOFMS was conducted in negative ion mode with the capillary voltage set at 3.5 kV. The flow rate of heated dry nitrogen gas (heater temperature, 300 °C) was maintained at 12 L/min and the nebulizer gas was set at 20 psi. The fragmentor, skimmer, and Oct RFV voltages were set at 100 V, 50 V, and 200 V, respectively. Each acquired spectrum was automatically recalibrated using an Agilent G1969-85001 API-TOF reference mass solution kit (*m*/*z* 112.9856 and *m*/*z* 1033.9881). Quantification was performed by comparing the D- and L-2HG peak areas to the calibration curve generated using internal standardization techniques with 2-morpholinoethanesulfonate.

### CE-TOFMS metabolite quantification

Intracellular metabolites were detected and quantified using CE-TOFMS (Agilent Technologies) as described previously [45–47]. Metabolite identities were assigned by matching their m/z values and migration times to standard compounds.

### Microarray analysis

Microarray analysis was conducted as described previously [48]. Total RNA was isolated from HT29 and HCT116 cells treated with or without 500 μM OC-L2HG for 24 h using the RNeasy Mini Kit (Qiagen, Venlo, Netherlands). RNA quality was assessed using an Agilent 2100 Bioanalyzer (Agilent Technologies). cRNA amplified from 100 ng total RNA was labeled using a Low Input Quick Amp Labeling Kit, One-Color (Agilent Technologies), hybridized to a SurePrint G3 Human GE 8x60K v2 microarray (Agilent Technologies) and then scanned using an Agilent scanner according to the manufacturer’s instructions. Relative hybridization intensity and background hybridization values were calculated using Agilent Feature Extraction Software (Agilent Technologies). Microarray data were analyzed using GeneSpring software (Agilent Technologies).

### Interactome analysis

Genes commonly altered in OC-L2HG-treated cells (upregulation: fold change (FC) > 2, downregulation: FC < 0.5) and CRC tissues (upregulation: false discovery rate (FDR) < 0.05, FC > 0, downregulation: FDR < 0.05, FC < 0) were extracted. The interactome tool in the MetaCore platform [49] was used to examine candidate transcription factors in the upregulated and downregulated gene sets.

### Hypoxic stress

To expose cells to hypoxic stress, they were cultured for 24 h in a modular incubation chamber (MIC-101, Billups-Rothenberg, San Diego, CA, USA) set to hypoxic conditions (1% O_2_).

### mTOR pathway analysis under serum deprivation

Cells were seeded in RPMI 1640 medium containing 10% FBS for 16 h and then moved to medium without FBS and pre-cultured for 24 h. The culture medium was then changed again to FBS-free medium and cells were treated with OC-L2HG, rapamycin, and/or Torin1 for 24 h.

### ATF4 immunocytochemistry

Cells were fixed with 4% paraformaldehyde (Thermo Fisher Scientific, Waltham, MA, USA) in phosphate buffered saline (PBS) for 15 min, rinsed with PBS, and permeabilized with 0.2% Triton X-100 (Nakarai Tesque) in PBS for 10 min. Cells were blocked using 1% goat serum (#16210–064, Thermo Fisher Scientific) in PBS for 15 min and incubated with ATF4 antibodies (1:100; #11815, Cell Signaling Technology, Denvers, MA, USA) diluted in 1% goat serum/PBS at 4 °C overnight. After rinsing in PBS, cells were incubated with Alexa Fluor 647–conjugated secondary antibodies (1:200, Thermo Fisher Scientific) and Hoechst 33342 (Thermo Fisher Scientific) as a nuclear stain (diluted to a final concentration of 1 µg/mL) for 2 h at 25 °C in the dark. Images of stained cells were obtained and the ATF4 intensity in Hoechst 33342-stained nuclei was measured using a Cytell Cell Imaging System (Cytiva, Preston, UK) with a 10× objective lens. All experiments were performed with three biological replicates.

### Luciferase reporter assay

Cells were co-transfected with pGL4-AAREx4-SV40-Luc2 (1 μg), a luciferase reporter plasmid containing four repetitions of the ATF4 binding motif (AARE) and the renilla luciferase plasmid pRL-TK (0.2 μg; Promega, Madison, WI, USA) as an internal control, and then treated with OC-L2HG, OC-D2HG, rapamycin, and/or Torin1 for 24 h. Firefly and Renilla luciferase signals were measured using a Dual-Luciferase Reporter Assay System (Promega) on a TECAN microplate reader using Magellan software (Tecan Group Ltd., Männedorf, Switzerland).

### Real-time PCR analysis

Real-time PCR analysis was conducted as described previously [50]. Briefly, RNA was isolated from cells using TRIzol reagent (Thermo Fisher Scientific) according to the manufacturer’s protocol and 2 μg of RNA was used for reverse transcription with a First Strand cDNA Synthesis kit (ReverTra Aceα; Toyobo Co., Osaka, Japan). Quantitative real-time PCR was carried out on a StepOne Plus Real-Time PCR system (Applied Biosystems, Foster City, CA, USA) using SYBR premix Ex Taq (Takara, Shiga, Japan) according to the manufacturer’s instructions. The ΔΔCq method was used to quantify gene expression, using RPL27 expression as an internal reference [51]. All experiments were performed in triplicate. The primers used for real-time PCR are described in Supplementary Table 3.

### siRNA transfection

siRNA duplexes for ATF4, L2HGDH, OGDH, and a negative control were purchased from Sigma-Aldrich. Cells were seeded in 6-well plates overnight and then transfected with 100 pmol of siRNA oligomer mixed with Lipofectamine RNAiMAX reagent (Thermo Fisher Scientific) in serum-reduced Opti-MEM (Thermo Fisher Scientific) according to the manufacturer’s instructions. After 4–5 h, complete culture medium was added to each well and cells were incubated at 37 °C in a CO_2_ incubator for another 24 h.

### Cell viability assay

Cell viability was measured using the 3-(4,5-dimethylthiazol-2-yl)-2,5-diphenyltetrazolium bromide (MTT, Sigma-Aldrich) assay or trypan blue exclusion assay. MTT assays were performed as described previously [52]. Briefly, cells (5 × 10^3^ cells/well) were seeded in each well of a 96-well plate and incubated for 24 h. Cells were treated with OC-D2HG or OC-L2HG under serum-free conditions for 2, 4, 6, and 8 d. Next, 50 μL MTT (2 mg/mL in PBS) was added to each well and plates were incubated for a further 2 h. After aspiration of the culture medium, the resulting formazan crystals were dissolved in 100 μL dimethyl sulfoxide (DMSO) and plates were placed on a plate shaker for 1 min before being read immediately at 570 nm using a TECAN micro-plate reader with Magellan software (Tecan Group Ltd.).

For the trypan blue exclusion assay, 2 × 10^5^ cells/well were seeded in 12-well cell culture plates and incubated at 37 °C. After treatment with reagents under serum-free conditions, cells were disaggregated in 500 μL medium and 10 μL of the suspension was mixed with 10 μL trypan blue (Thermo Fisher Scientific). Viable cells were counted using a Countess Automated Cell Counter (Thermo Fisher Scientific).

### Western blotting

Immunoblot analysis was performed as described previously [48] using primary antibodies against GAPDH (1:4000; 10494-1-AP, Proteintech, MA, USA), actin (1:5000; sc-47778, Santa Cruz Biotechnology, Inc., CA, USA), cleaved PARP (1:1000; #5625, Cell Signaling Technology), cleaved caspase-3 (1:1000; #9664, Cell Signaling Technology), LC3B (1:1000; #2775, Cell Signaling Technology), ATF4 (1:1000; #11815, Cell Signaling Technology), phospho-p70 S6 kinase (Thr389) (1:1000; #9234, Cell Signaling Technology), p70 S6 kinase (1:1000; #2708, Cell Signaling Technology), phospho-AKT (Thr308) (1:1000; #9275, Cell Signaling Technology), phospho-AKT (Ser437) (1:1000; #4060, Cell Signaling Technology), AKT (1:1000; #4691, Cell Signaling Technology), phospho-4E-BP1 (Thr37/46) (1:1000; #2855, Cell Signaling Technology), phospho-4E-BP1 (Ser65) (1:1000; #9451, Cell Signaling Technology), phospho-4E-BP1 (Thr70) (1:1000; #9455, Cell Signaling Technology), 4E-BP1 (1:1000; #9644, Cell Signaling Technology), DEPTOR (1:1000; #11816, Cell Signaling Technology), L2HGDH (1:1000; #15707-1-AP, Proteintech), V5 Tag (1:1000; #37-7500, Thermo Fisher Scientific), and LSD1 (1:1000; #2184, Cell Signaling Technology), and horseradish peroxidase-conjugated secondary antibodies (Cytiva).

### Vector construction

For shRNA expression, control shRNAs targeting LacZ had the following sequence: 5′-gcuacacaaaucagcgauuucgaaaaaucgcugauuuguguagc-3′. The shRNAs against L2HGDH had the following sequences: 5′-caccagctcauuugauauagcgaacuauaucaaaugagcuggugc-3′ (#1) and 5′-ccuuuaaacgagaggguuaccgaaguaacccucucguuuaaaggc-3′ (#2). Targeted gene sequences were subcloned as deoxyribose fragments into pENTR/U6 TOPO (Thermo Fischer Scientific) and recombined into the lentivirus vector pLenti6 BLOCKiT. Lentiviral vectors were generated and used according to the manufacturer’s instructions.

For DOX-inducible expression vectors, L2HGDH cDNA with a BamHI restriction site at the 5′ end and an EcoRI restriction site at the 3′ end was synthesized by Eurofins Japan (Tokyo, Japan) and subcloned into the pRetroX-TetOne vector (TaKaRa) using the BamHI and EcoRI restriction sites. Retroviral vectors were generated and used according to the manufacturer’s instructions.

### Analysis of correlations between amino acids and 2HG levels in human CRC tumors and adjacent non-neoplastic tissues

We reanalyzed the levels of amino acids and 2HG in paired tumors and adjacent non-neoplastic tissues from 275 CRC patients using our previously reported metabolomic data [18]. The correlations between amino acids and 2HG levels were analyzed using Spearman’s correlation.

### Statistics and reproducibility

We performed all experiments at least twice and confirmed similar results. Statistical analyses were performed using GraphPad Prism v8.0 software (GraphPad Software, Inc., La Jolla, CA, USA). For in vitro experiments, data from two or more groups were analyzed using Student’s *t*-tests and one-way analysis of variance (ANOVA), respectively. For in vivo experiments, data from two or more groups were analyzed using Mann-Whitney U and Kruskal-Wallis tests, respectively. The correlations between amino acids and 2HG levels in Fig. 8 were analyzed using Spearman’s correlation. Data are represented as mean ± SEM or ±SD; *P* values < 0.05 were considered statistically significant.

## Supplementary information

Supplementray figures

Supplementray table 1

Supplementray table 2

Supplementray table 3

## Data Availability

Metabolome data are included in Supplementary Data. Microarray data were deposited in the National Center for Biotechnology Information GEO with the accession code GSE210167. All other data will be available upon reasonable request.
