# Supplementary material for: L-2hydroxyglutaric acid rewires amino acid metabolism in colorectal cancer via the mTOR-ATF4 axis
Source: Oncogene. 2023 Mar 6;42(16):1294–307. doi: 10.1038/s41388-023-02632-7 (PMC10101855; doi:10.1038/s41388-023-02632-7)
Supplement: Supplementary file 1 — Supplementray figures [file 41388_2023_2632_MOESM1_ESM.pdf]

Supplementary Fig. S1

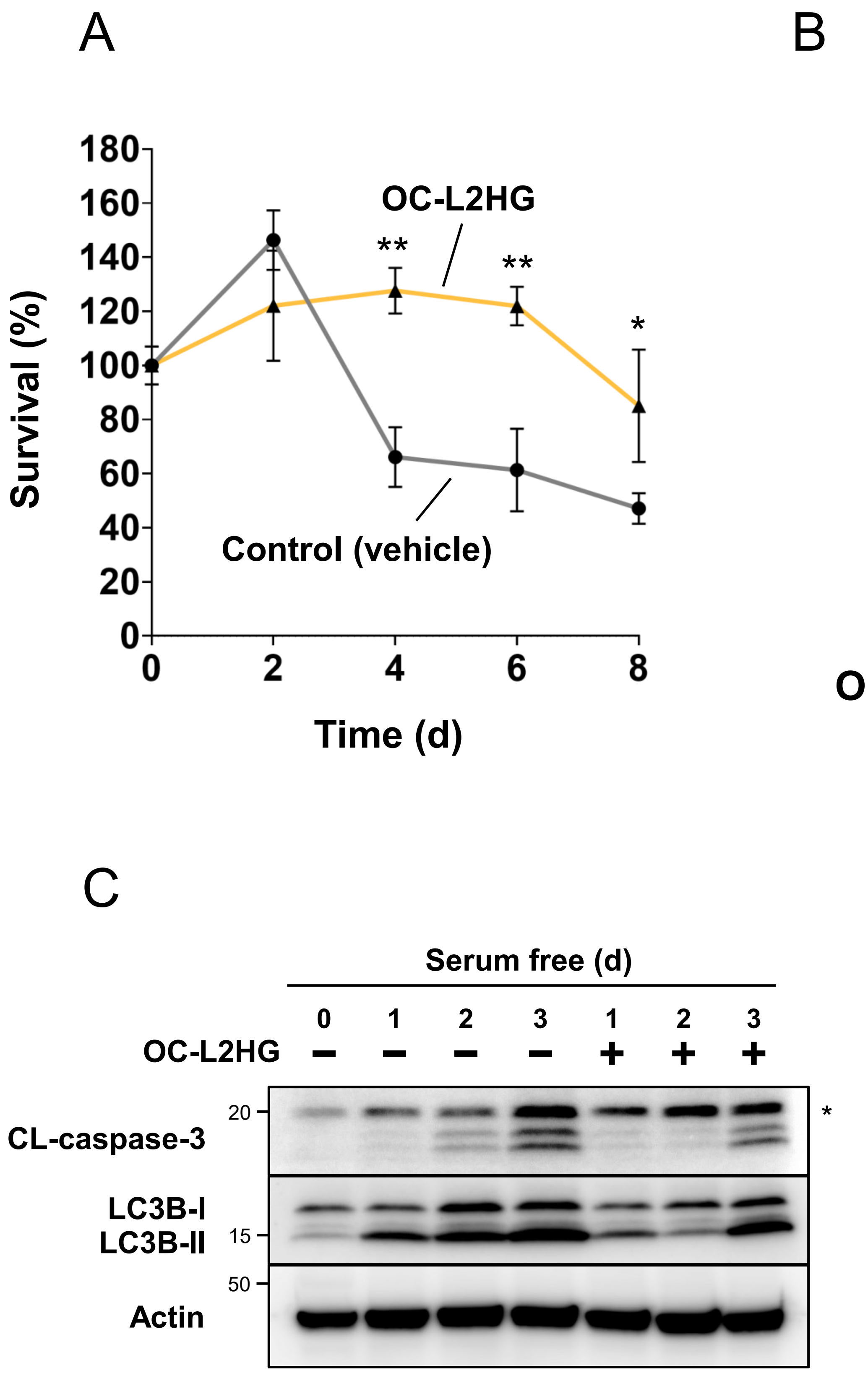

Supplementary Fig. S1. L2HG levels are increased in colorectal tumors (related to Figure 1).
(A) Viability in HT29 cells treated with OC-L2HG (250 μM) under serum-free conditions. Viability was assessed using MTT assays. Data are presented as the mean ± SD of three independent experiments. * $P < 0.05$ and ** $P < 0.01$. (B) Viability in CaR1 cells treated with OC-L2HG (250 μM) under serum-free conditions for 6 d. Viable cells were counted using a trypan blue exclusion assay. Data represent the mean ± SD of three independent experiments. (C) Expression of CL-caspase 3 and LC3B in CaR1 cells treated with OC-L2HG under serum-free conditions. Asterisk, nonspecific band.

Supplementary Fig. S2

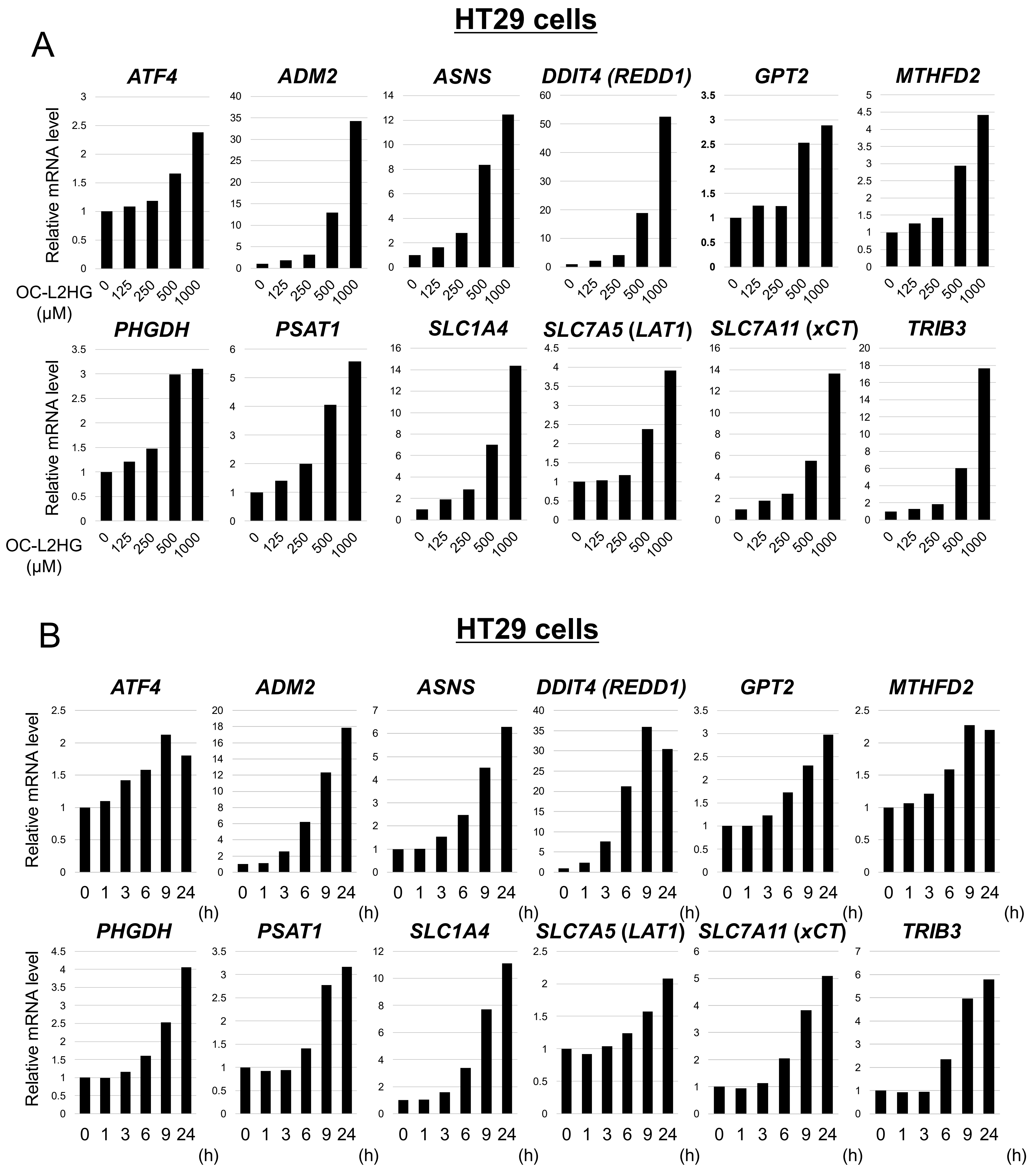

HTCT116 cells

C

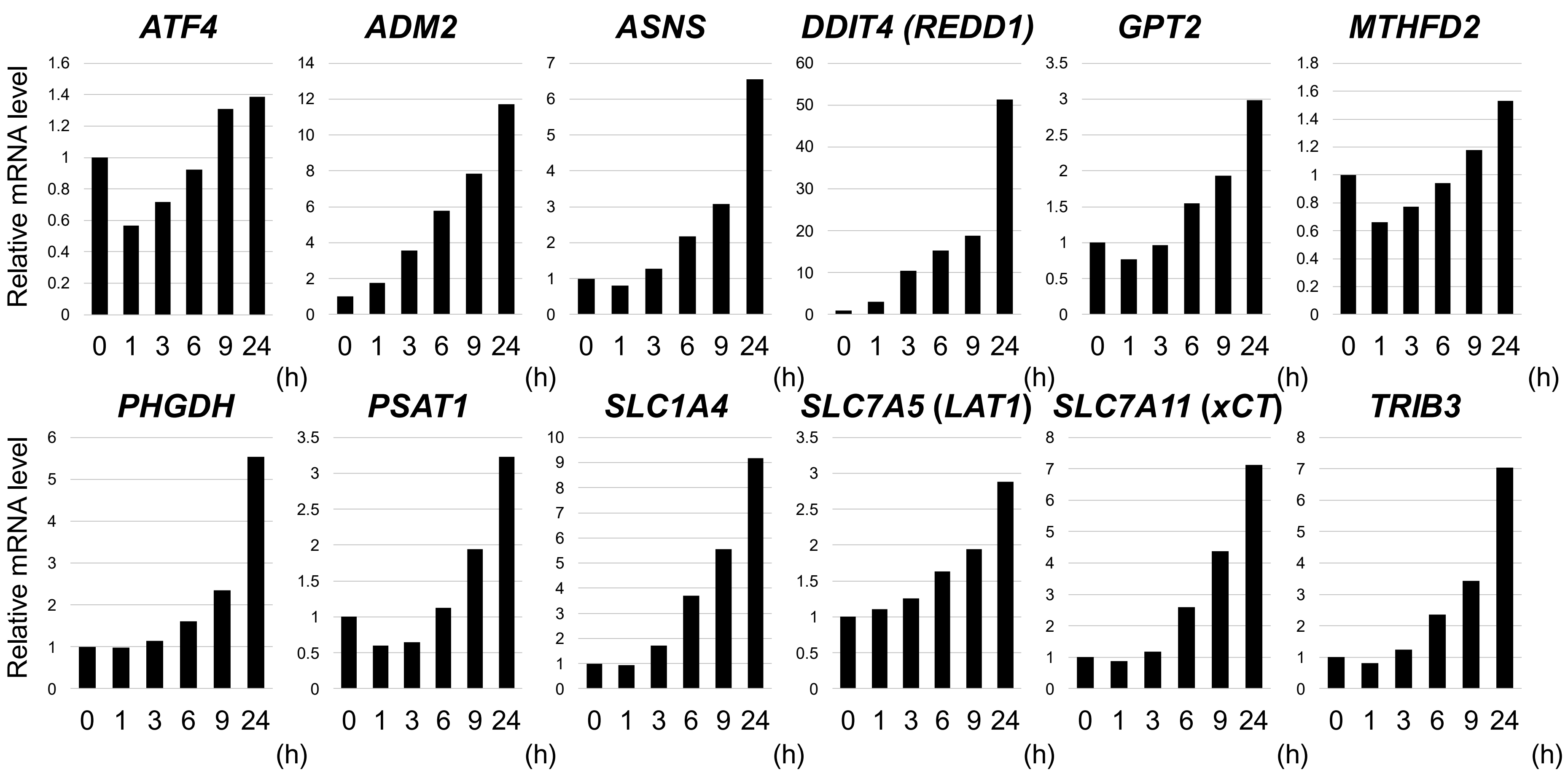

D

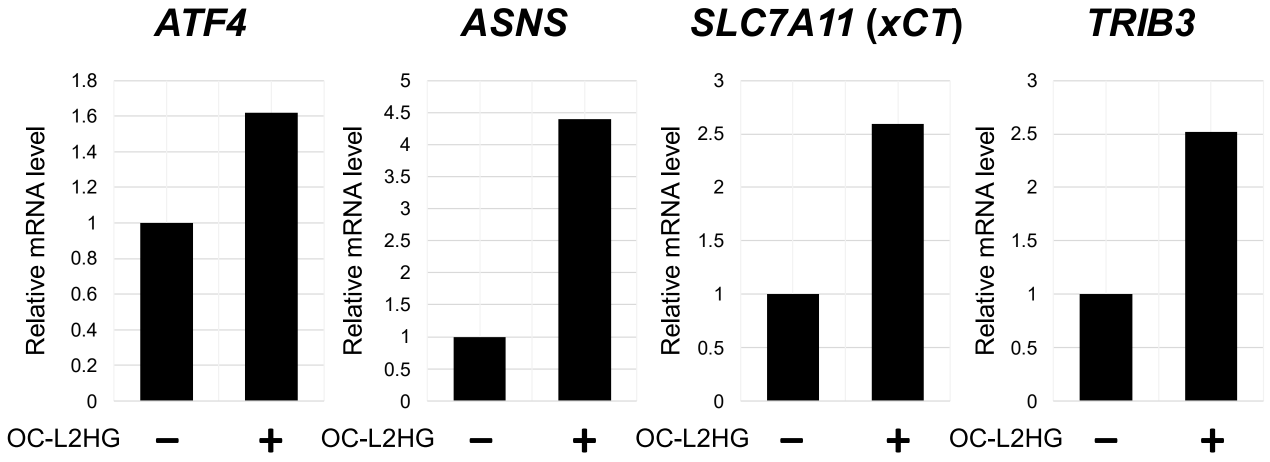

E

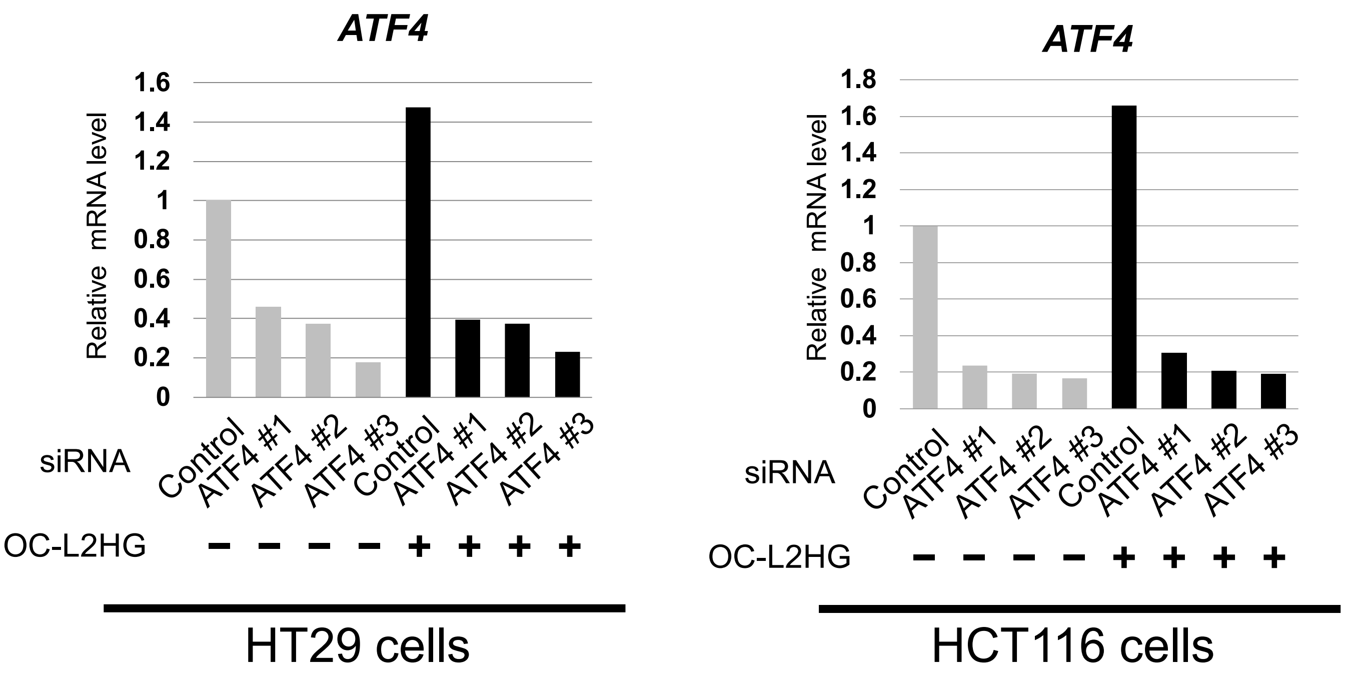

F

HT29 cells

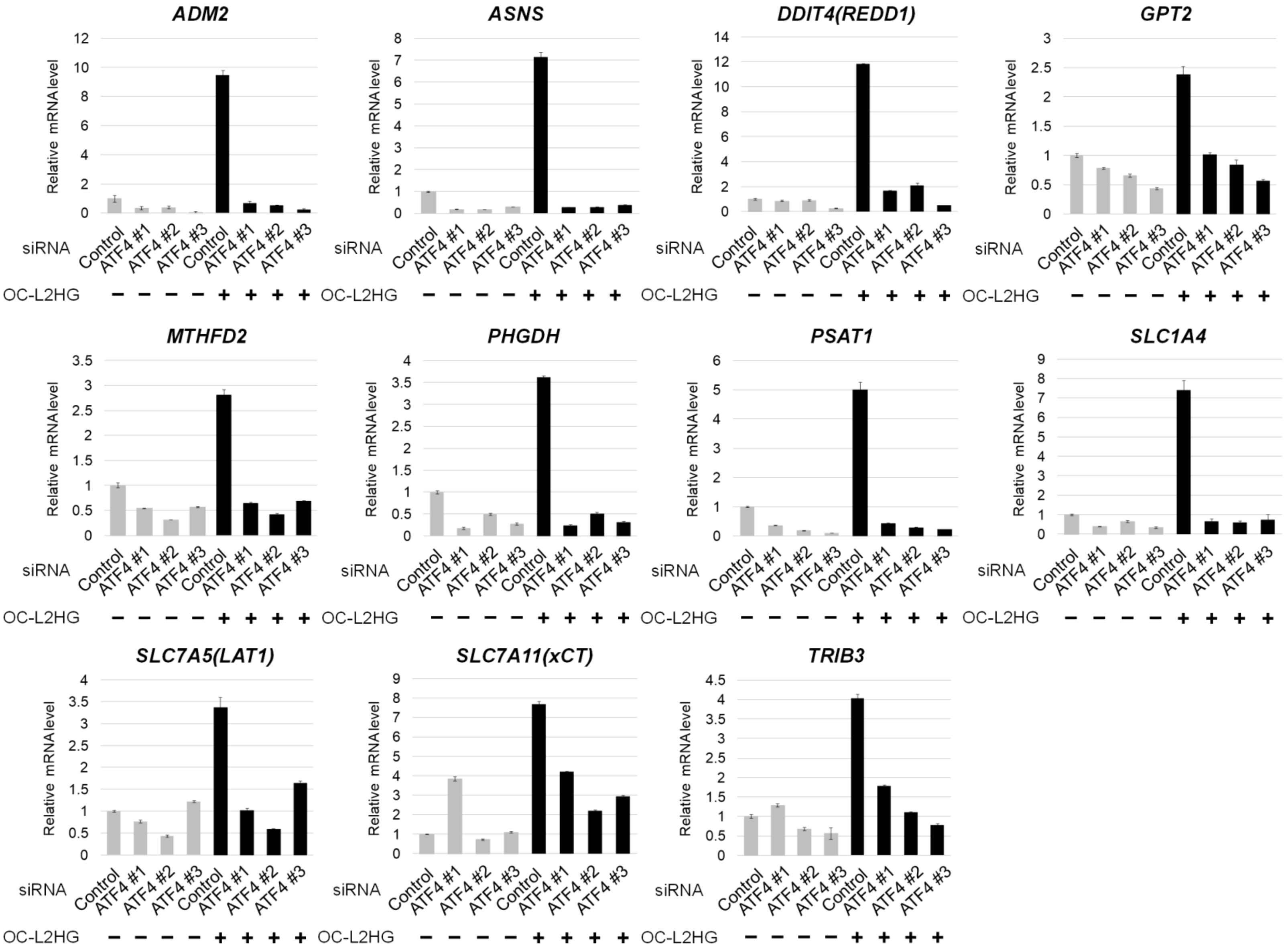

HTCT116 cells

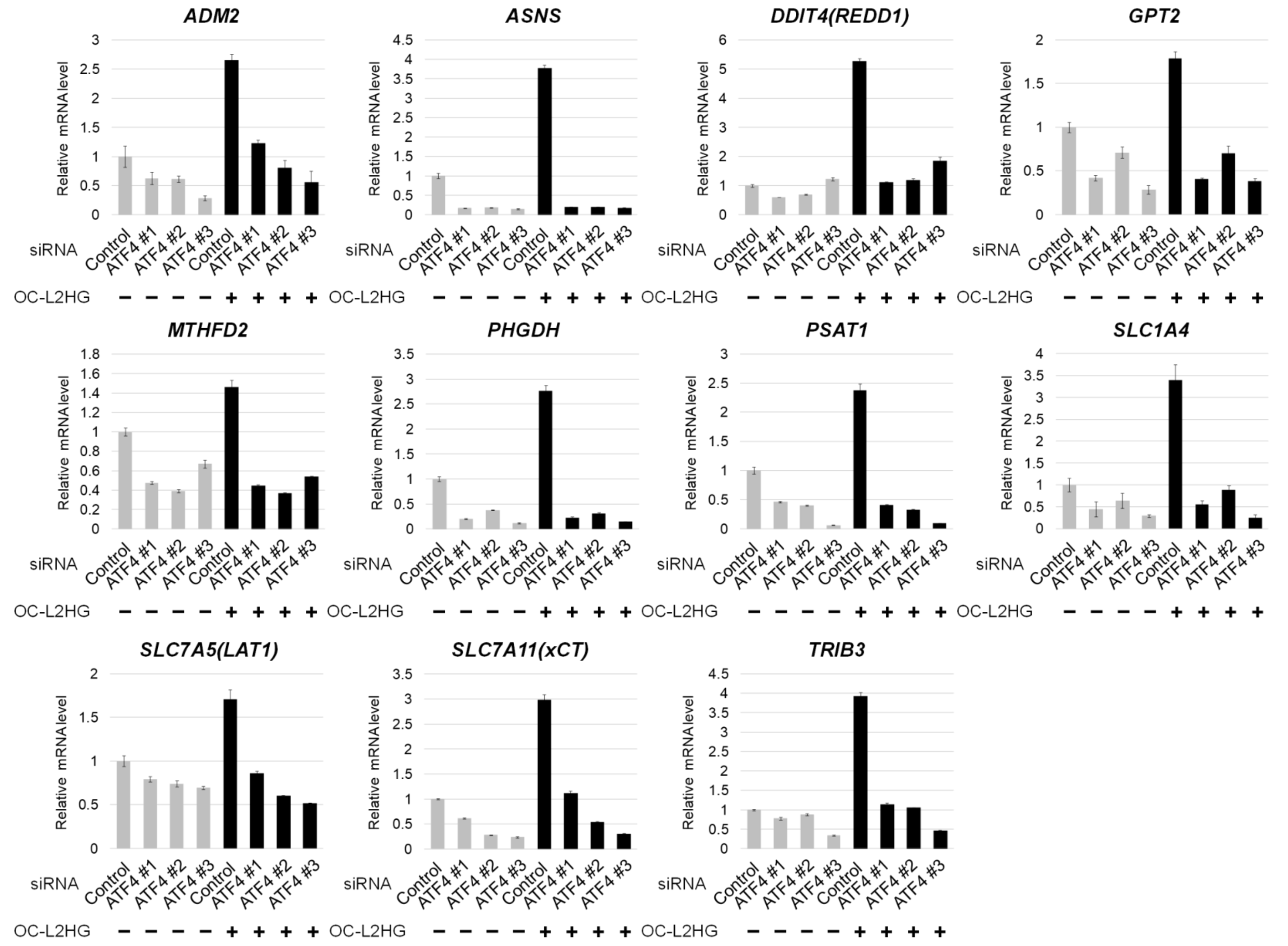

G

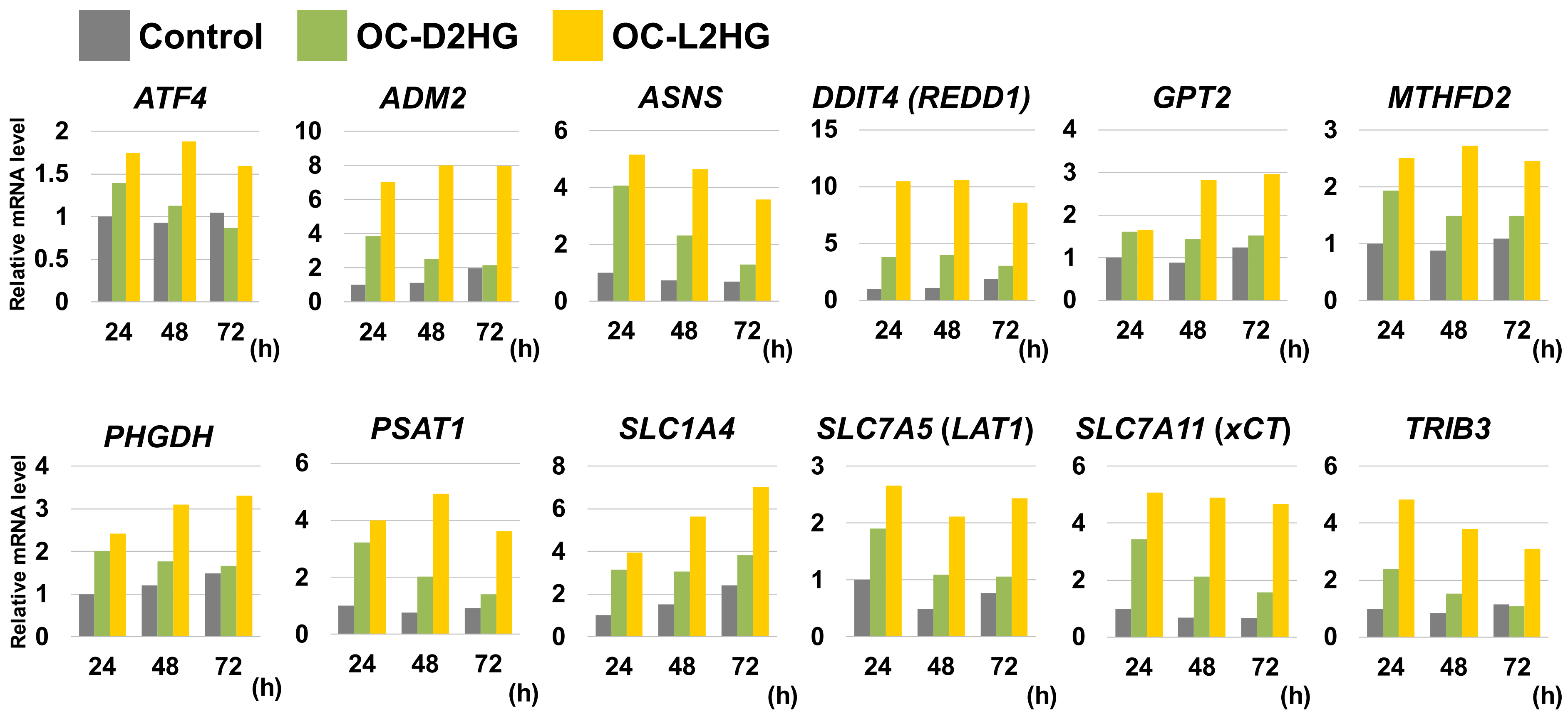

Supplementary Fig. S2. L2HG induces ATF4 activation in CRC cells (related to Figure 2).

(A) mRNA expression of ATF4 and ATF4 target genes in HT29 cells treated with OC-L2HG for 24 h in a dose-dependent manner. (B) mRNA expression of ATF4 and ATF4 target genes in HT29 cells treated with 500 μM OC-L2HG in a time-dependent manner. (C) mRNA expression of ATF4 and ATF4 target genes in HCT116 cells treated with OC-L2HG for 24 h in a dose-dependent manner. (D) mRNA expression of ATF4 and ATF4 target genes in CaR1 cells treated with 500 μM OC-L2HG for 24 h. (E) siRNA knockdown of ATF4 in HT29 and HCT116 cells treated with and without 500 μM OC-L2HG for 24 h. (F) Effect of ATF4 siRNA on ATF4 target genes in HT29 and HCT116 cells treated with and without 500 μM OC-L2HG for 24 h. qPCR data are the average of 3 technical replicates and representative of at least 2 independent experiments with similar results. (G) Analysis of the mRNA expression of ATF4 target genes in HT29 cells stimulated with 500 μM OC-L2HG or 500 μM OC-D2HG for 24 h, using real-time PCR. Data represent the average of three technical replicates and are representative of at least two independent experiments with similar results.

Supplementary Fig. S3

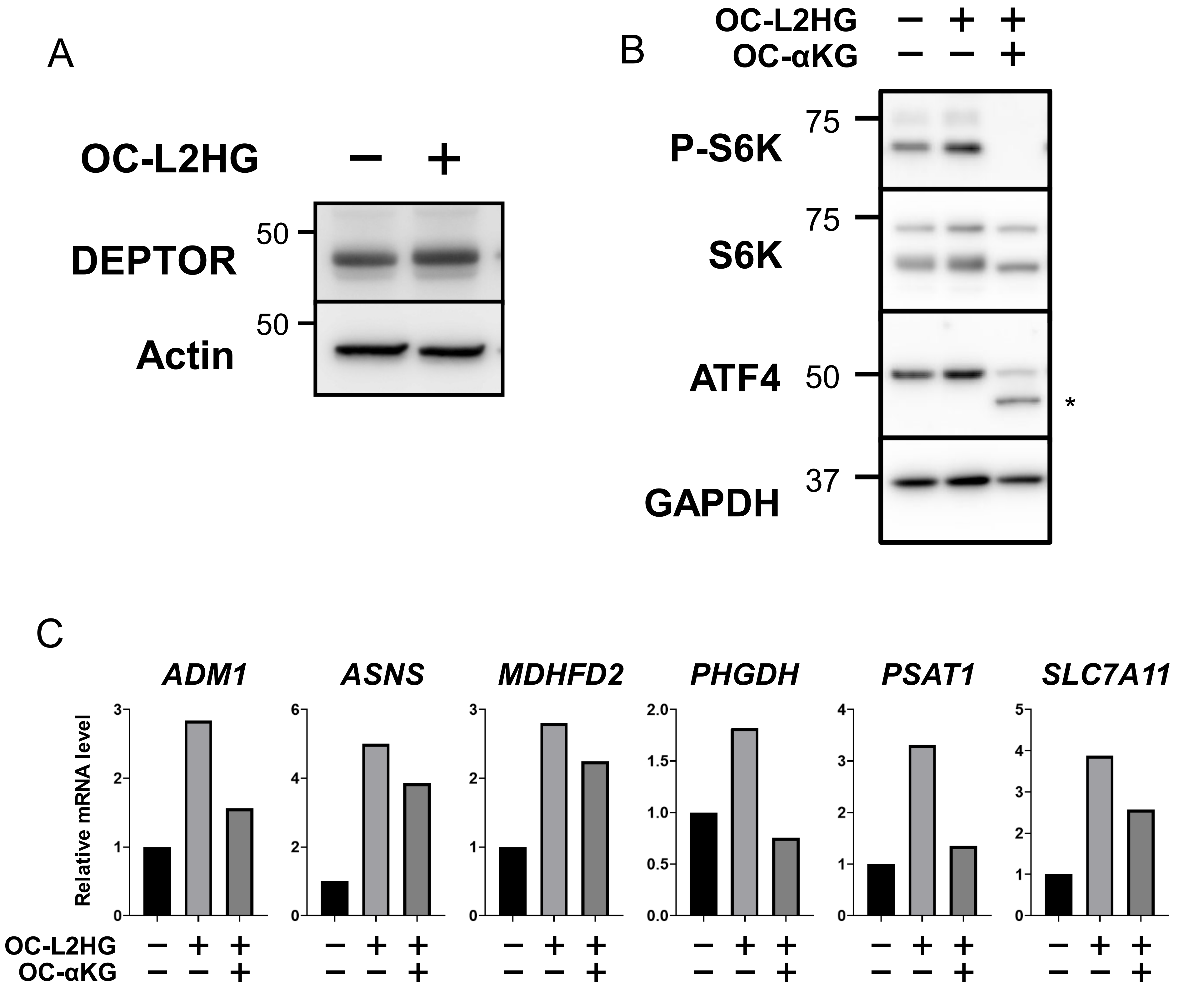

Supplementary Fig. S3. L2HG increases ATF4 expression via mTOR signaling in CRC cells (related to Figure 3).

(A) Expression of DEPTOR in HT29 cells treated with 500 μM OC-L2HG for 24 h under serum-free conditions. (B) Effect of OC-αKG (500 μM) on P-S6K and ATF4 expression in HT29 cells treated with OC-L2HG for 9 h under serum-free conditions. Asterisk, nonspecific band. (C) Effect of OC-αKG on the expression of ATF4 target genes in HT29 cells treated with OC-L2HG for 9 h under serum-free conditions.

Supplementary Fig. S4

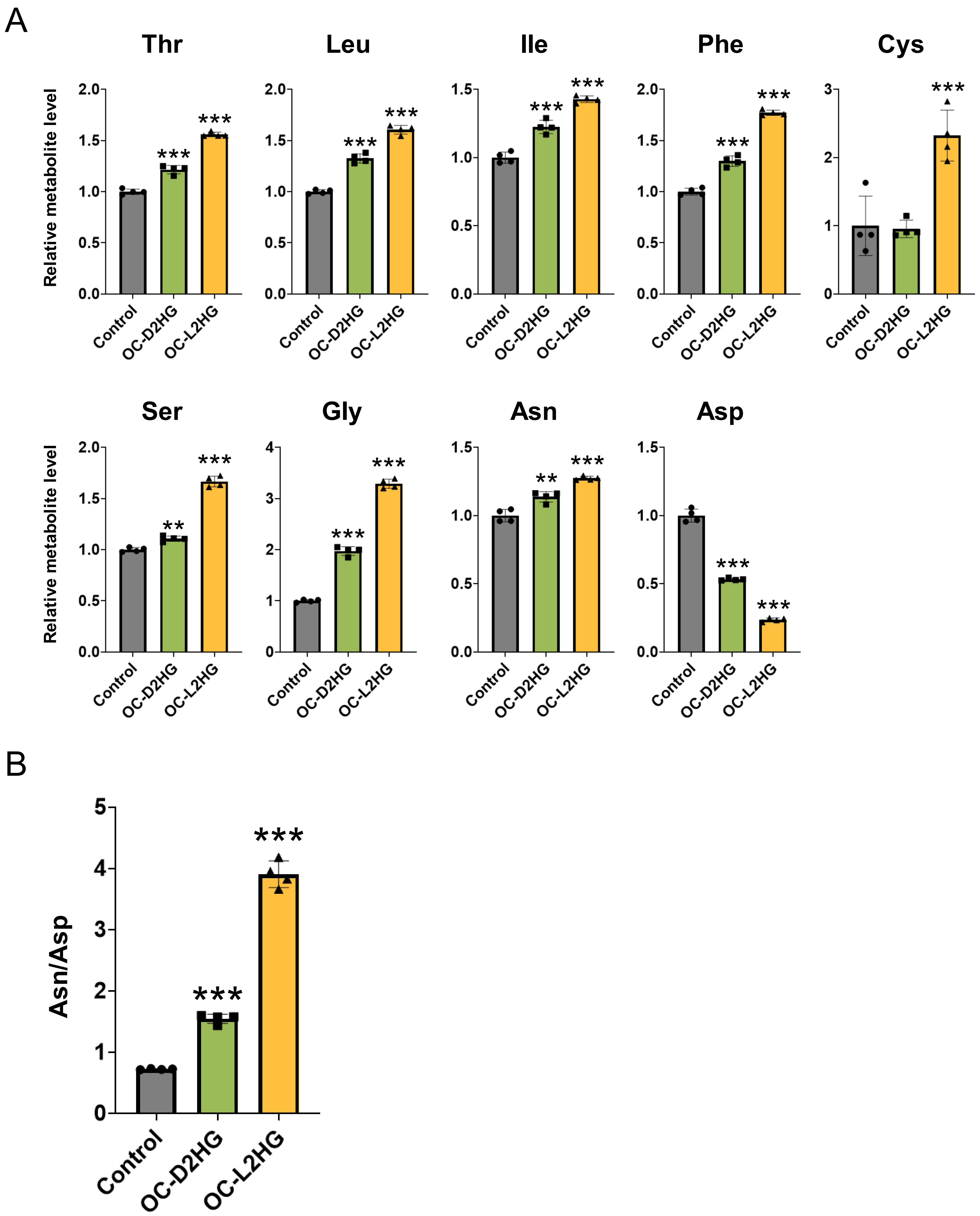

Supplementary Fig. S4. L2HG mediates ATF4 induction to regulate amino acid metabolism in CRC cells (related to Figure 4).

(A) Effect of OC-L2HG and OC-D2HG on the level of amino acids in HT29 cells. (B) Ratio of Asn to Asp in HT29 cells treated with OC-L2HG and OC-D2HG. Data are presented as the mean ± SD of three independent experiments. ** $P < 0.01$ and *** $P < 0.001$.

Supplementary Fig. S5

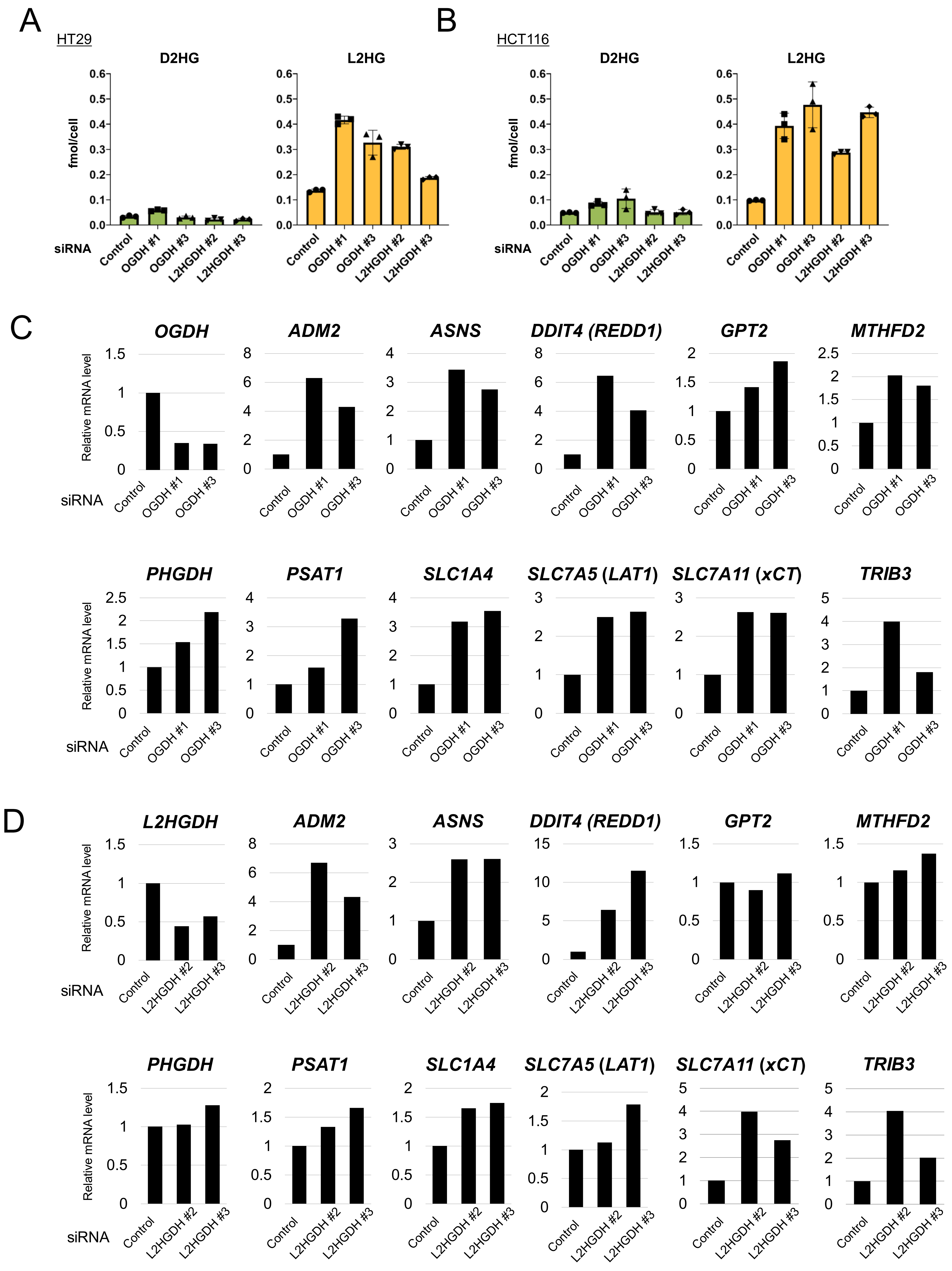

Supplementary Fig. S5. L2HGDH and OGDH downregulation increase L2HG levels and induce ATF4 in CRC (related to Figure 5).

(A and B) Levels of D/L2HG in HT29 cells (A) and HCT116 cells (B) transfected with OGDH and L2HGDH siRNA. Data are presented as the mean ± SD of three independent experiments. (C) Effect of OGDH knockdown on mRNA expression of OGDH and ATF4 target genes in HT29 cells, using real-time PCR. qPCR data are the average of 3 technical replicates and representative of at least 2 independent experiments with similar results. (D) Effect of L2HGDH knockdown on mRNA expression of L2HGDH and ATF4 target genes in HT29 cells.

Supplementary Fig. S6

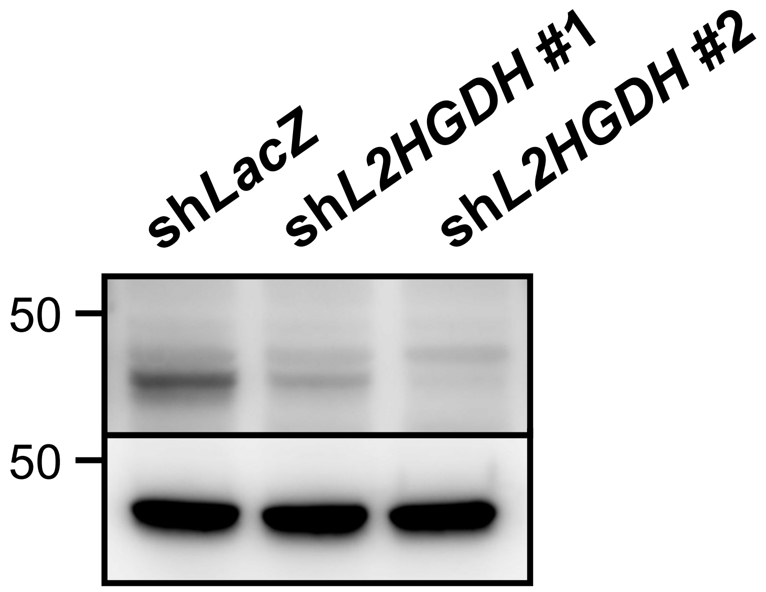

Supplementary Fig. S6. L2HGDH knockdown increases tumor growth and alters amino acid metabolism *in vivo* (related to Figure 7).
shRNA-mediated knockdown of L2HGDH in HT29 cells.
